# *In vitro* evaluation of efficacy of different rotary instrument systems for gutta percha removal during root canal retreatment

**DOI:** 10.4317/jced.52488

**Published:** 2016-10-01

**Authors:** Mercy Joseph, Jyoti Ahlawat, Amit Malhotra, Murali Rao, Abhimanyu Sharma, Sangeeta Talwar

**Affiliations:** 1 MDS. Conservative Dentistry and Endodontics, D.A.P.M.R.V. Dental College, Bangalore, India; 2 Senior Resident. Department of Conservative Dentistry and Endodontics, Maulana Azad Institute of Dental Sciences, New Delhi, India; 3 Professor. Department of Conservative Dentistry and Endodontics, D.A.P.M.R.V. Dental College, Bangalore, India; 4 MDS. Oral and Maxillofacial Surgery, ESIC Hospital, Rohini, New Delhi, India; 5 Professor. Department of Conservative Dentistry and Endodontics, Maulana Azad Institute of Dental Sciences New Delhi, India

## Abstract

**Background:**

Complete removal of old filling material during root canal retreatment is fundamental for predictable cleaning and shaping of canal anatomy. Most of the retreatment methods tested in earlier studies have shown inability to achieve complete removal of root canal filling. Therefore the aim of this investigation was to assess the efficacy of three different rotary nickel titanium retreatment systems and Hedstrom files in removing filling material from root canals.

**Material and Methods:**

Sixty extracted mandibular premolars were decoronated to leave 15 mm root. Specimen were hand instrumented and obturated using gutta percha and AH plus root canal sealer. After storage period of two weeks, roots were retreated with three (Protaper retreatment files, Mtwo retreatment files, NRT GPR) rotary retreatment instrument systems and Hedstroem files. Subsequently, samples were sectioned longitudinally and examined under stereomicroscope. Digital images were recorded and evaluated using Digital Image Analysing Software. The retreatment time was recorded for each tooth using a stopwatch. The area of canal and the residual filling material was recorded in mm2 and the percentage of remaining filling material on canal walls was calculated. Data was analysed using ANOVA test.

**Results:**

Significantly less amount of residual filling material was present in protaper and Mtwo instrumented teeth (*p* < 0.05) compared to NRT GPR and Hedstrom files group. Protaper instruments also required lesser time during removal of filling material followed by Mtwo instruments, NRT GPR files and Hedstrom files.

**Conclusions:**

None of the instruments were able to remove the filling material completely from root canal. Protaper universal retreatment system and Mtwo retreatment files were more efficient and faster compared to NRT GPR fles and Hedstrom files.

** Key words:**Gutta-percha removal, nickel titanium, root canal retreatment, rotary instruments.

## Introduction

Long term success of endodontic therapy relies on thorough debridement of the root canal system followed by three dimensional obturation. Inability to achieve these goals results in persistence of intracanal pathogens, eventually leading to treatment failure. Non surgical retreatment is often considered the treatment of choice in management of failed endodontic cases with a success rate of 74 to 98 % (1,2). During retreatment procedure, complete removal of root canal filling material is of utmost importance in order to achieve effective cleaning and disinfection of canal anatomy (3).

Although various obturation materials have been introduced in recent years, however Gutta percha in combination with root canal sealer still appears to be the most commonly used material (4). Various methods available for removal of root canal filling material include the use of hand files, rotary instruments, heat, ultrasonics, laser and adjunctive use of solvents (5,6). Removal of gutta percha with manual instrumentation is a tedious and time consuming procedure (7). Therefore, in order to allow effective removal of filling material and shorten the treatment time, various rotary nickel titanium retreatment instrument systems have been introduced over the last decade (1). Various chemicals that have been used as gutta percha solvents include chloroform, eucalyptol, xylene, halothane, turpentine. In the current study, eucalyptol was used as a solvent since it has been reported to be safe and non carcinogenic.

The aim of the current study was to evaluate the efficiency of different rotary instrument systems - Protaper Universal system (Dentsply Maillefer, Ballaigues, Switzerland), MTwo Retreatment system (Sweden and Martina, Padova, Italy) and a new NRT GPR file system (Mani Inc., Japan), compared with manual instrumentation with H-files for gutta percha removal during retreatment.

The ProTaper universal retreatment files are characterised by progressively increasing tapers, a convex triangular cross section and a modified guiding tip. They consist of three instruments (D1, D2, D3) with various tapers and diameters at the tip (size 30, 0.09 taper, size 25, 0.08 taper, size 20, 0.07 taper). D1 file has an active tip that aids in facilitating penetration of subsequent files. The non-active tips of D2 and D3 reduce the incidence of ledging, perforation and stripping during removal of filling materials (3). Mtwo Retreatment files have an S-shaped cross-section and two cutting edges. These files have a shorter pitch length which may enhance advancement of the file into the filling material (1).

A relatively new rotary file system for gutta-percha removal, namely the NRT GPR has been manufactured by Mani Inc., which has helical grooves along the working section. It is available in 4 sizes; 1S, 2S, 3N and 4N. 1S (size 70, 16 mm length, 0.04 taper) and 2S (size 50, 18 mm length, 0.04 taper) are stainless steel files which are used for gutta percha removal from the cervical and middle third of the canal. The 3N (size 40, 21 mm length, 0.04 taper) and 4N files (size 30, 21 mm length, 0.04 taper) are Ni Ti files that are used till the working length.

## Material and Methods

Sixty extracted Mandibular premolars with single root canal were selected. Soft tissue and calculus were mechanically removed from the root surfaces. The current investigation is an *in vitro* study where extracted teeth were used without any intervention/experimentation in human subjects. Necessary clearance and approval was obtained from the ethics committee.

After completing the access cavity preparation, size 10 K file (Dentsply Maillefer, Ballaigues, Switzerland) was inserted in the root canal until it was just visible at the apical foramen. 1 mm was subtracted from this measurement to calculate the working length. The samples were decoronated subsequently to achieve a standard size of 15 mm.

-Initial root canal treatment:

Canal instrumentation was performed with K-files in a step back fashion to size 30 at working length, stepping back with three subsequent instruments [35,40,45]. Final coronal flaring was done with Gates Glidden drills (GG drill) size 2 and 3. Irrigation was done between successive instruments with 5 ml sodium hypochlorite (2.5 %) delivered with 30-gauge needle tips (NaviTip, Ultradent, South Jordan, UT, USA). On completion of instrumentation, canals were rinsed with EDTA (17 %) for 3 minutes, followed by final irrigation with 5 ml of sodium hypochlorite. After drying the canal with sterile paper points, obturation was done with gutta percha and AH plus sealer (Dentsply De Tray, Konstanz, Germany) using thermomechanical compaction in a hybrid technique. 8 In this technique, gutta percha was compacted laterally in the apical region of the canal, followed by use of a rotating Gutta Condenser (size 35, 8000 rpm, Dentsply-Maillefer) to heat soften the filling material in coronal portion of canal. The quality and extent of root fillings were evaluated with digital radiographs. Subsequently the access cavities were sealed with Cavit (ESPE, Dental Seefeld, Germany). The specimens were stored in an incubator at 37 degree centigrade in 100% humidity for 4 weeks to allow the sealer to set completely.

-Retreatment techniques:

All specimen were retreated by a single operator. Teeth were randomly divided into four groups of fifteen specimen each (n=15). Temporary restorations were removed with size 4 round bur (Mani Dia Bur). Eucalyptol was used as a solvent during retreatment procedures. Canals were irrigated with sodium hypochlorite (2.5 %) using a 30-gauge needle after each instrument change. All instruments were discarded after use in five root canals. Retreatment was considered complete when no remnants of gutta percha and sealer were observed on instrument surface or in the irrigating solution. Careful inspection of canal anatomy and instrument surface was done using magnifying loupes 4.5x (Carl Zeiss, Oberkochen, Germany). Retreatment time was calculated using a stopwatch.

-Group I : Hedstrom files

Filling material was removed from the coronal portion of canal with GG drill size 2 and 3. Thereafter a drop of solvent was placed in the canal. Hedstrom files (Dentsply Maillefer) of size 35, 30, 25 were used to remove root fillings in circumferential quarter turn push-pull motion until working length was reached. Apical preparation was done H file to size 40.

-Group II: Protaper Universal Retreatment Files 

Protaper retreatment files were used at a speed of 600 rpm in a brushing action against the canal walls. Filling material from the coronal portion of canal was removed with D1 file (size 30, 0.09 taper). D2 protaper file (size 25, 0.08 taper) was inserted till the middle third followed by D3 (size 20, 0.07 taper) at the working length. Apical preparation was done with finishing files F2 (size 25,0.08 taper), F3 (size 30,0.09 taper) and F4 (size 40, 0.06 taper).

-Group III: Mtwo Retreatment files

Instruments were operated at speed of 600 rpm. Mtwo R2 instrument (size 25, 0.05 taper) was used till the working length. Final apical enlargement was done with Mtwo instrument of size 40, 0.04 taper.

-Group IV: NRT GPR gutta percha remover

Coronal gutta-percha was removed with GG drill, followed by deposition of eucalyptol for 2 min. Retreatment was performed using 2S (size 50, 18 mm length, 0.04 taper) and 4N (size 30, 21 mm length,0.04 taper) instruments against the canal walls in a crown down fashion until working length was reached.

**Figure 1:**
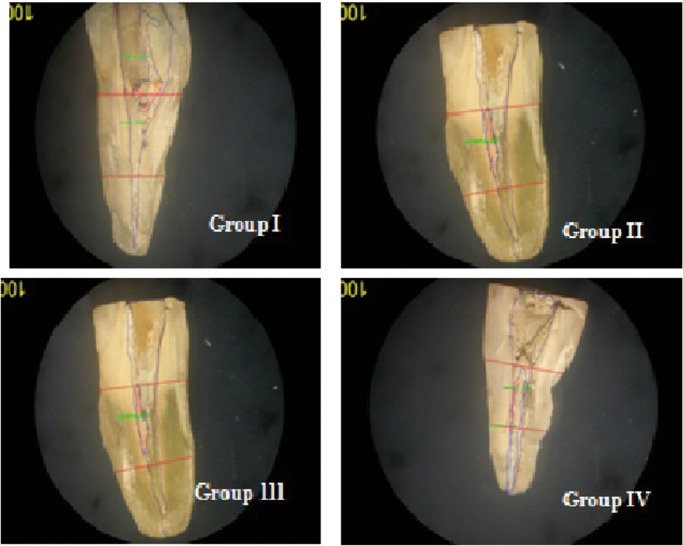
Assessment of residual gutta percha using image pro-express 6.0 software.

-Analysis of residual gutta percha:

Roots were grooved longitudinally using a diamond disk, preparing grooves parallel to the long axis of buccal and lingual surfaces, and split using a rongeur into halves. Sections that showed evidence that the groove had penetrated into the root canal space or exhibited an irregular cleavage were discarded, replaced by a new specimen. Samples were examined under a stereomicroscope at 12.5X magnification. After being photographed with a digital camera, the images were evaluated using Digital Image Analysing Software, Image-pro Express 6.0 (Media cybernetics) (Fig. 1). For practical purposes no attempt was made to differentiate between gutta percha and sealer remnants. Removal was considered complete for all groups when no filling material was observed on instruments and no filling material was detected inside the canal using stereomicroscope. The retreatment time was recorded for each tooth using a stopwatch. The residual filling debris was outlined by a single operator blinded to group assignment. The area of the canal and the residual filling material was recorded in mm2 and the percentage of remaining filling material on canal walls was calculated with the following equation: Area of remaining filling material X 100 = Area % of remaining filling material

-Area of canal wall

Data was analysed using ANOVA test in order to compare the percentage of remaining filling material after retreatment. Significance level was set at *p* < 0.05.

## Results

**Figure 2:**
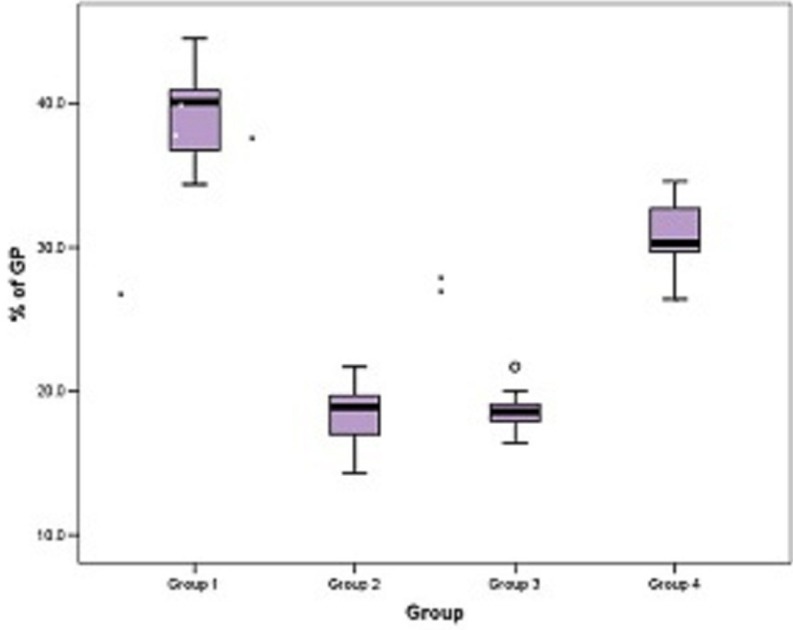
Box plot showing mean % of total gp remaining in the entire tooth among the four groups.

Remnants of filling material was observed in all groups. Analysis of the overall means of percentages of residual filling material showed significantly less values for Protaper universal retreatment instruments and Mtwo retreatment instruments (*p*< 0.05), followed by NRT GPR gutta percha removal instruments and hedstrom files respectively ( Table 1, Fig. 2).

**Table 1:**
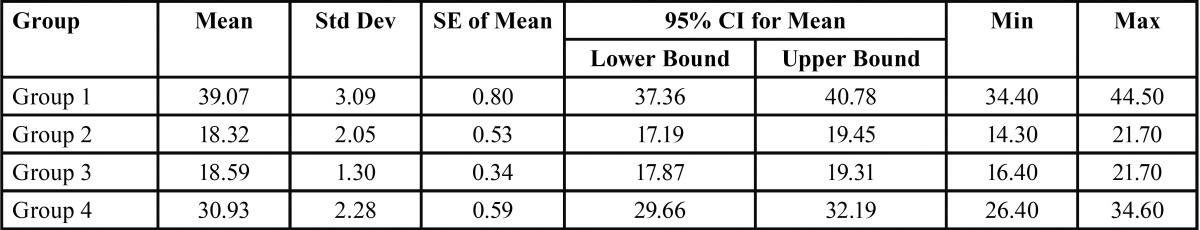
Comparison of the mean % of total gp remaining on the entire root canal wall.

**Figure 3:**
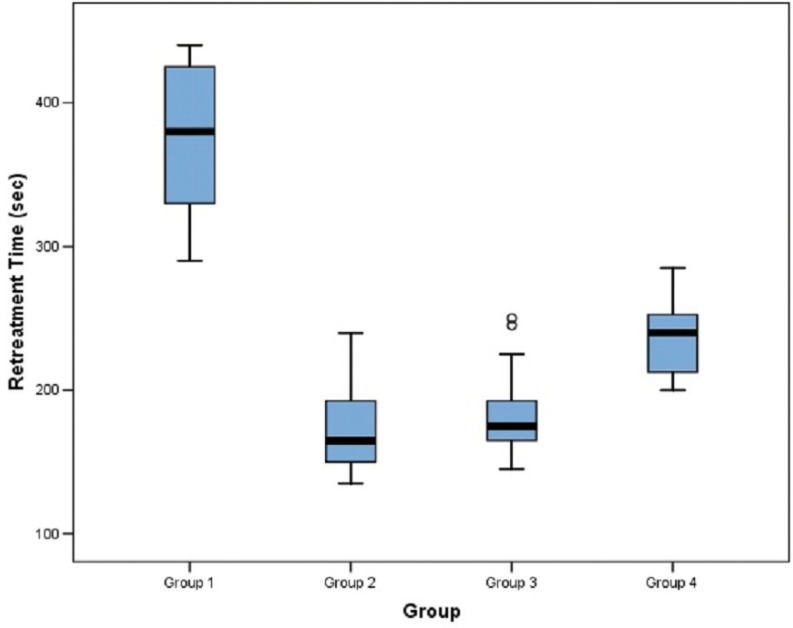
Box plot comparison of retreatment time (in sec) among the four groups.

Time taken for GP removal and reinstrumentation was also least for Protaper group followed by Mtwo instruments, NRT GPR files and hedstrom files respectively ( Table 2, Fig. 3).

**Table 2:**
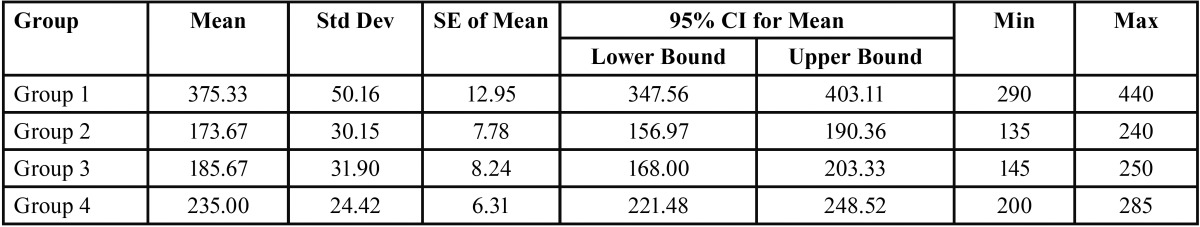
Comparison of retreatment time (in sec) among the four groups.

## Discussion

The most crucial step during non surgical retreatment is thorough removal of canal filling material which allows further instrumentation and disinfection of the canal system (5). Various studies have demonstrated increased efficacy and safety of NiTi rotary instruments for root canal preparation. Cleaning and shaping with rotary NiTi instruments is associated with minimal risk of alteration of canal anatomy and reduced treatment time compared to hand instruments (9). Teeth were decoronated to allow standardization of samples. Various methods that have been employed earlier to evaluate the efficacy of removal of filling material. The residual material inside the root canal has been assessed by radiographs, making the teeth transparent , longitudinal splitting of the teeth (10,11). Current investigation involved longitudinal sectioning of the samples followed by observation under stereomicroscope at 12.5x magnification. Microphotographs were recorded with a digital camera and subsequently analysed using Digital Image Analysing Software, Image Pro Express version 6.0, a software which analyses the area in each third of the canal space in mm2. Although numerous investigators have used scoring criterias to calculate the amount of residual filling material on canal surface, however digital analysis using the above mentioned software may seem to be a more precise and accurate approach (12).

None of the retreatment techniques were able to remove all the filling material, a finding that is consistent with previous studies (13,14). Retreatment was performed in significantly less time with the rotary instruments than the manual technique which is in agreement with earlier reports (3,4). On evaluation of total percentage of residual filling material protaper instruments showed maximum efficacy, followed by Mtwo group and NRT GPR group. Teeth retreated with H files demonstrated highest percentage of residual gutta percha. Comparison of mean retreatment time also showed significantly less treatment time required by Protaper retreatment files and Mtwo retreatment instruments compared to NRT GPR files and hand instrumentation.

Various authors have speculated that rotary instruments are less efficient than hand instruments in GP removal (4,10,15-17). A possible reason for this could be the failure to perform apical enlargement beyond initial preparation size during retreatment procedure. Further apical preparation is desirable since the tip diameter of last instrument provided in rotary retreatment system (Protaper Retreatment file, size 20; R2 Mtwo file, size 25) is ineffective in performing adequate cleaning and shaping in apical region (18,19). In the current study, apical enlargement was done till size 40 in Protaper and Mtwo groups, which resulted in significantly cleaner canal walls. The use of solvents during retreatment procedure is also controversial since they might contribute to incomplete removal of filling material by softening the gutta percha and leaving behind a residual film on root canal walls (20). Such a scenario was taken into consideration in the current study, whereby solvent was used only in the coronal portion of canal in order to facilitate initial penetration of instruments in gutta percha and minimal volume of solvent was employed during the procedure.

The findings of current study demonstrated favourable outcome for Protaper retreatment system which is similar to the results of investigations carried out by Guiliani *et al.* and Takahashi *et al.* (15,17) Guiliani *et al.* attributed the gutta percha removal ability of Protaper universal retreatment instruments to the spirals running around the instruments and the negative cutting angle which produces cutting action instead of planning the gutta percha against the canal walls (15). Retreatment occurs faster with NiTi rotary files compared to hand instrumentation because of plasticization or softening of gutta percha by action of rotary instruments, thus leading to easier removal of material (4,21). Samples instrumented with Protaper retreatment files showed less amount of filling material left inside the canal compared to Mtwo files, although the difference was not statistically significant. These findings are consistent with those reported by Bramante *et al.* The authors attributed the rapid and efficient performance of Protaper retreatment instruments to their higher taper and more metallic core compared to Mtwo files. Such a design of working blade causes increased heat release and rapid plasticization of gutta percha (21,22).

A new gutta percha remover, NRT GPR manufactured by Mani Inc., Japan was used in this study. The advantages of this system is that it is hard to fracture around tip part since there is no concentration of stresses due to non-cutting tip. If they do fracture, N3 and N4 will fracture at the neck, and is easy to extract from the canal. However, an important drawback of these instruments is limited cutting efficacy compared to other retreatment systems available currently. In addition, its minimum tip size being #30, does not penetrate into apical gutta percha where the apical part is prepared to sizes lesser than #30. Original NRT rotary instruments had a limitation of decreased flexibility, resulting in inability to go around canal curvatures (23). This may also hold true in case of NRT retreatment files which compromises GP removal efficacy. Current findings showed lesser gutta percha removal efficacy of these instruments along with prolonged treatment time, compared to Protaper and Mtwo instruments.

Since the current investigation utilised teeth with straight root canals, such findings cannot be correlated directly in cases of teeth with curved canal morphology especially when the safety provided by rotary instruments is taken into account regarding fractures and procedural errors. An investigation carried out by Beasley *et al.* demonstrated fractures of few D3 files in Protaper Universal Retreatment system while attempting removal of filling material in moderately curved root canals. Increased taper and the speed recommended by manufacturer for operating these files could result in excessive torsional fatigue of the instrument during use, resulting in fracture or deformation (24). Therefore, further studies need to be carried out to explore the efficiency of rotary nickel titanium instruments in retreatment procedure of teeth with curved or abrupt canal morphology.

## Conclusions

None of the retreatment systems were successful in removing all the filling material inside the canal. With regard to cleaning efficacy as well as treatment duration, protaper instruments showed best results followed by Mtwo and NRT GPR system. Present investigation also outlined the importance of enlarging the apical preparation size during re-instrumentation, in order to achieve thorough cleaning and shaping of canal anatomy.
